# An integrated risk assessment model for indoor air quality impacts on museum exhibits and human health using microclimatic indices and AgNPs treatment

**DOI:** 10.1371/journal.pone.0336594

**Published:** 2025-12-10

**Authors:** Alexandru Ilieș, Tudor Caciora, Gabriela Ilieș, Thowayeb H. Hassan, Zharas Berdenov, Dorina Camelia Ilieș, Ana Cornelia Pereș, Bahodirhon Safarov, Vasile Grama, Horvath Sabina, Liliana Indrie, David Mínguez-García, Raquel Belda-Anaya, Pablo Diaz-Garcia

**Affiliations:** 1 Department of Geography, Tourism and Territorial Planning, Faculty of Geography, Tourism and Sport, University of Oradea, Oradea, Romania; 2 Faculty of Geography, Babes-Bolyai University of Cluj-Napoca, Sighetul Marmatiei Extension, Romania; 3 Social Studies Department, College of Arts, King Faisal University, Al Ahsa, Saudi Arabia; 4 Faculty of Science, L.N. Gumilyov Eurasian National University, Nur-Sultan, Kazakhstan; 5 Department of Environmental Engineering, Faculty of Environmental Protection, University of Oradea, Oradea, Romania; 6 Department of Digital Economy, Samarkand Branch of Tashkent State University of Economics, Samarkand, Uzbekistan; 7 Țării Crișurilor Museum, Calea Armatei Române 1/A, Oradea, Romania; 8 Department of Textiles, Leather and Industrial Management, Faculty of Energy Engineering and Industrial Management, University of Oradea, Oradea, Romania; 9 Universitat Politècnica de València, Departamento de Ingeniería Textil y Papelera, Alcoi, Spain; Changan University: Chang'an University, CHINA

## Abstract

The quality of indoor air in museums directly influences the conservation of exhibits made of organic materials, but also the health of visitors, employees and restorers. Thus, monitoring the microclimate and identifying non-invasive solutions to limit its impact becomes essential for the protection of movable cultural heritage. Thus, this study aims to monitor ten indicators of indoor air quality and their impact on exhibits, visitors and employees in a museum in Romania. The data obtained were analysed using specific indexes to accurately assess the indoor microclimate risk for both human health and the preservation of exhibits. The values obtained in terms of the indexes certify the stability in terms of the quality of the indoor air. Subsequently, six ethnographic fabric samples were tested to evaluate cleaning and preservation solutions based on silver nanoparticles, aiming to reduce microbial contamination and ensure sustainable protection of organic museum textiles. The results obtained indicate a moderate risk of degradation of exhibits, mainly caused by the increased relative humidity, which exceeded the limits recommended by the international standards in force. As for human health, the applied indices show that the indoor environment does not threaten human health in general, but it still records non-compliant values of the formaldehyde indicator. Also, ionic imbalance shows poor ventilation, associated with potential discomfort and negative cognitive effects. The application of silver nanoparticle treatment has proven effective in reducing microbiological contamination, providing a sustainable conservation solution, both immediately after application and after 30 days. The study highlights the importance of constant monitoring and control of environmental parameters to ensure optimal conditions for the conservation of material heritage and to prevent risks to human health in museum spaces.

## 1. Introduction

The microclimate parameters, such as temperature (T) and relative humidity (RH) can induce significant threats to organic materials used in movable cultural heritage such as textiles, wood and leather, because unstable conditions can speed up chemical, biological and mechanical degradation processes [1–2]. Thus, the monitoring of indoor air quality (IAQ) stands as a critical preventive conservation strategy for cultural heritage protection according to Ilies et al. [3], Ruga et al. [4], Marchetti et al. [5] and Ilies et al. [6] especially for museum collections [7–10]. The specific IAQ parameters together with bacteriological and fungal microflora can directly affect the physicochemical stability of exhibited materials as demonstrated by Bernardi and Camuffo [11], Camuffo et al. [12] and Bonacina et al. [13]. Organic materials undergo rapid biological degradation even following minor changes in environmental parameters, which affects both the physical condition and the historical value of the objects. The maintenance of controlled microclimate conditions serves both as a technical necessity and as a fundamental requirement to protect museum collections from deterioration [14].

In addition to the continuous determination of IAQ and the identification of possible exceedances of the international standards in force for certain parameters, it is also of real importance to identify solutions for the protection of the exhibits. The specialized literature currently considers the use of non-invasive materials to protect the exhibits without affecting their physical and mechanical integrity [15,16]. Some natural dyes used in textiles have antibacterial properties, which is why they are increasingly being studied for preservation purposes. Thus, alizarin inhibits bacteria such as *E. coli* and *P. aeruginosa* by affecting the cell walls [17,18]. Indigo has demonstrated antimicrobial activity even in the absence of light, against pathogens such as *S. aureus* and *C. albicans* [19–21]. Turmeric, due to its chemical structure, provides coloring and antimicrobial protection to textile fibers [22–25]. Walnut and onion husks also add both color and antibacterial effect against Gram-positive and Gram-negative bacteria [26–28]. Recent studies consider the use of nanoparticles for cleaning materials in order to preserve them for a longer period of time [29,30]. Nanoparticles represent a modern, efficient and less invasive alternative compared to traditional cleaning substances used in the conservation of cultural heritage, especially with regard to textiles. Due to their nanometric size and high chemical compatibility, these materials allow delicate interventions that do not compromise the physicochemical structure of the restored objects. Unlike conventional methods that can lead to irreversible damage, the use of nanoparticles minimizes the risks of alteration and provides increased durability to the treatments applied [31–33].

In addition to the risk that IAQ induces in terms of the conservation of cultural heritage exhibits inside museums, it can also have a major influence on human health, both of visitors and of employees and restorers [34]. Thus, some researches approach the issue of IAQ from a dual perspective, in the sense that the microclimatic parameters inside historical buildings have been monitored at determined time intervals, being subsequently analyzed and reported both from the perspective of the risk of deterioration of buildings and heritage objects, and from the perspective of the impact on public health [35,36]. This aspect is individualized against the background of the finding that prolonged exposure to such an indoor environment, characterized by polluting factors, can favor the emergence of conditions or the aggravation of pre-existing ones, particularly among vulnerable categories, such as children, the elderly or people with chronic diseases [37–39].

In accordance with the above, the objective of this study is to assess the IAQ in Țării Crișurilor Museum (Oradea, Romania), focusing on the spaces housing the ethnographic collection, in order to analyze the impact on the conservation of organic exhibits and on the health of visitors and staff. The study involves monitoring essential microclimate parameters, such as T, RH, CO₂, particulate matter (PM), formaldehyde (HCHO), total volatile organic compounds (TVOC), the amount of positive (I^+^) and negative (I^-^) ions, artificial brightness (AL) and the application of risk indexes to assess the conservation conditions of the exhibits and safety for human health. At the same time, the research includes testing microbiological contamination on textile samples and applying an antimicrobial treatment with silver nanoparticles (AgNPs). It is assumed that certain values of environmental indicators may exceed the permissible limits, negatively affecting the exhibits, and the treatment with nanoparticles can effectively reduce microbiological risks on organic materials. The novelty of the study is the dual approach regarding IAQ monitoring, the application of IAQ indexes to accurately determine the risks to which exhibits and people are exposed, as well as the practical validation of nanoparticle treatments on real exhibits within the museum.

The research gap identified is the limited number of studies that integrate, in a unified framework, the simultaneous assessment of risks affecting both material heritage and human health, correlated with the validation of innovative risk reduction approaches, such as AgNPs treatments applied directly to real museum objects. Furthermore, most existing research is limited to short-term monitoring campaigns or small collections, without proposing replicable and intelligent microclimate management models applicable to heritage buildings.

By applying a systematic air quality and microclimate monitoring system, based on integrated indices and microbiological contamination verification and cleaning techniques, this study proposes a reproducible approach for the management of heritage buildings, with a focus on museums.

## 2. Case study

**Fig 1:**
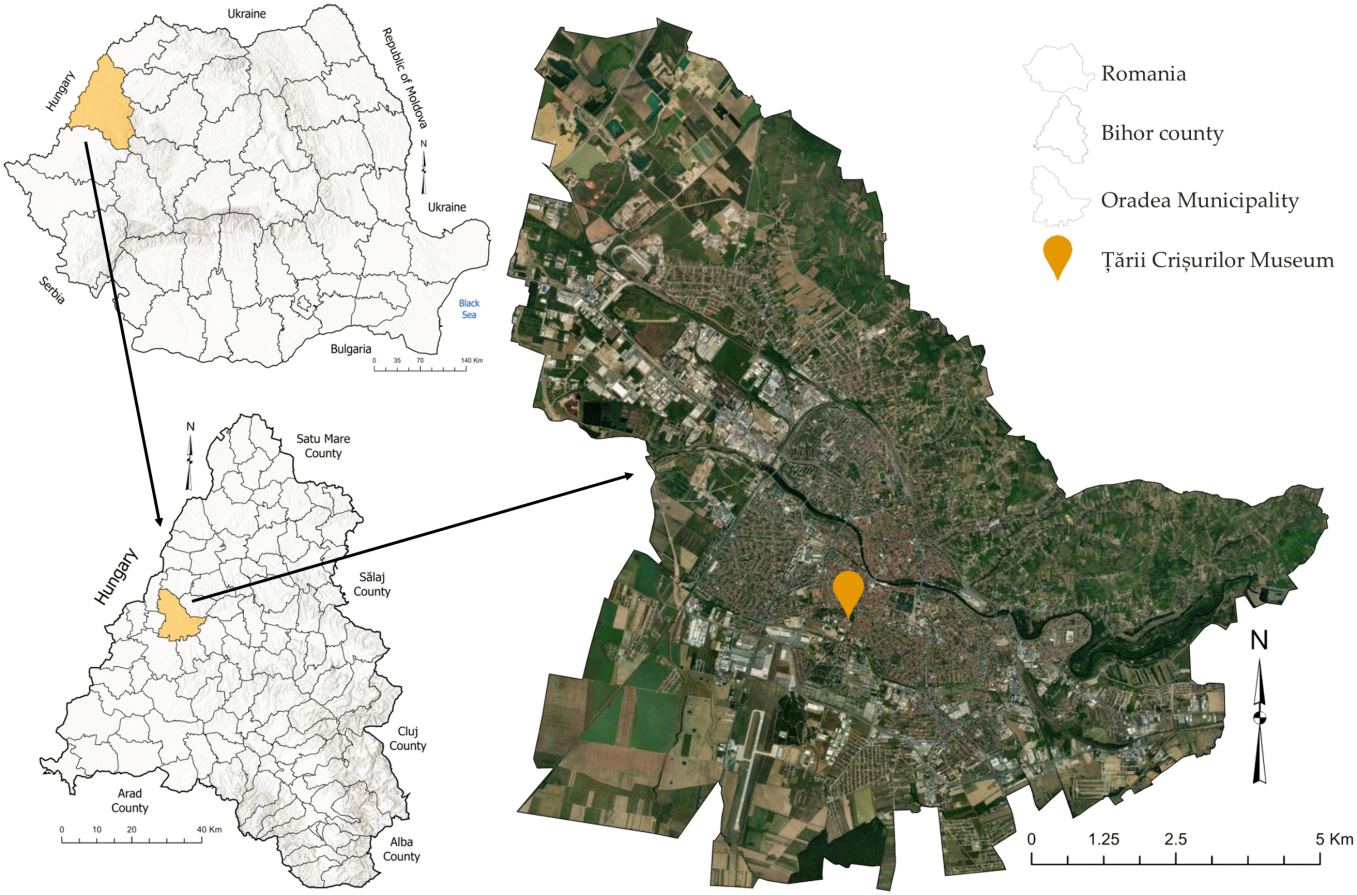
The location of the Țării Crișurilor Museum in Oradea Municipality, Romania *(Satellite imagery source: USGS EarthExplorer, ESRI World Imagery).*

The Țării Crișurilor Museum is located on Armatei Romane Street no. 1A, Oradea Municipality, Romania. It was opened in 1971 and is one of the largest pavilion museums in Romania. It currently holds a museum collection of approximately 450.000 archaeological, historical, ethnographic and art objects, representative for Western Romania, populated over time by Romanians, Swabians, Hungarians, Slovaks and other communities; this makes the objects held very diverse. Since 2006, the museum has been relocated to the former cadet school, a historic monument building arranged on four levels, built in 1897, whose main purpose was to serve military education. The Țării Crișurilor Museum has five permanent collections: natural sciences, archaeology, history, ethnography and art. In addition to these, there are temporary exhibitions that complete the cultural offer. The building is arranged on four levels and, in 2023, recorded a total number of 160.403 visitors, confirming its role as a reference cultural institution in western Romania. From a microclimatic point of view, the exhibition spaces rely exclusively on heating, ventilation, and air conditioning (HVAC) systems to ensure ventilation, as the architectural configuration does not include windows that allow natural ventilation. This structural feature has direct implications on the quality of indoor air, both for the comfort of visitors and for the conservation of the heritage objects on display (Fig 1).

For the present study, we chose to monitor the ethnography collection of the Țării Crișurilor Museum in terms of IAQ and microbiological contamination. The choice is motivated by the fact that the objects in the ethnography collection, especially wood and metal, ceramics, folk costumes, textiles, icons and religious objects and painted eggs, are extremely sensitive to variations in T, RH and other pollutants. These conditions can accelerate degradation processes, which is why it is essential to evaluate the interior conditions and their impacts. Unlike other collections, such as archaeology or art, which may benefit from additional protection through display cases or special storage conditions, many ethnographical exhibits are showcased directly in museum halls. This makes them more vulnerable to pollutants such as PM, RH and VOC variations. At the same time, the ethnography collection is one of the most accessible to the public, which means that it is more exposed to environmental factors, pollutants brought by visitors’ clothes and microbiological contamination.

Within this collection, three rooms were monitored. The first two communicate with each other through a permanently open door, consequently, we analyzed them as a unit. These two rooms have a total area of 78.7 m^2^ and a total air volume of 370 m^3^. The third room is characterized by a total surface area of 37.2 m² and an overall air volume of 175 m³. All three rooms analyzed share common characteristics, as they are exhibition spaces displaying traditional clothing and furniture items to the public. They are part of the same visiting route, so in a normal program, there are no differences in the number of visitors between them.

## 3. Materials and methods

**Fig 2:**
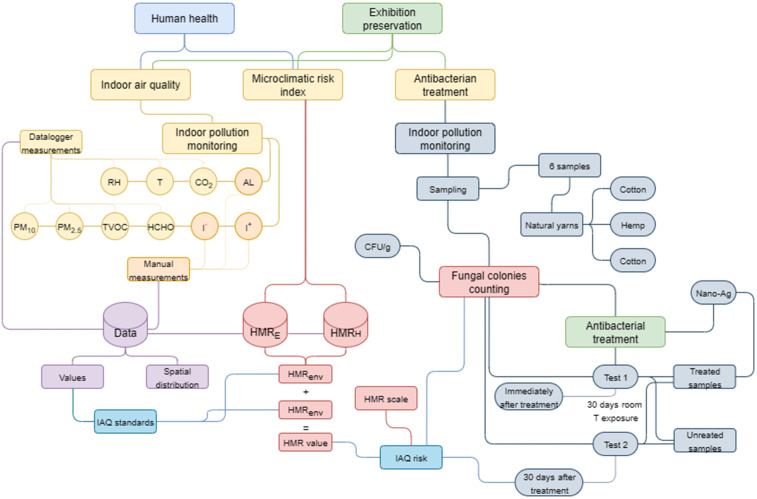
Diagram representing the work stages and methods used to achieve this objective from a scientific point of view.

For the determination of the potential risks to the conservation of exhibits and to the health of visitors and employees, a microclimate risk index, Heritage Microclimate Risk (HMR), was developed and applied, adapted for two major dimensions: HMR for the conservation of exhibits (HMR_E_) and HMR for human health (HMR_H_). The application of these indexes allows the quantification of the impact of indoor microclimate variables (T, RH, CO₂, PM, HCHO, TVOC, I^+^, I^-^, AL) on the integrity of the material heritage and on the sanitary comfort in the museum spaces (Fig 2).

Given the specific nature of the ethnographic collection, predominantly composed of organic materials such as textile fibers and wood, a microbiological analysis was also carried out on selected exhibits. Based on the degree of fungal contamination identified, antimicrobial treatments with AgNPs were applied, in order to evaluate their effectiveness as a long-term preservation method. Preliminary results suggest a significant reduction in bacterial and fungal load in the treated samples, highlighting the potential of using nanomaterials in ethnographic heritage protection strategies.

### 3.1. The determination of the indoor air quality

The data collection campaign spanned 8 months, from January to August 2024, when monitoring the values of 10 IAQ indicators was considered, namely: T, RH, CO_2_, I^+^, I^-^, HCHO, TVOC, PM_2.5_, PM_10_ and AL. After the monitoring period, all data were analyzed in relation to the international standards in force for determining IAQ, taking into account both human health and the conservation of exhibits (Fig 2).

**Fig 3:**
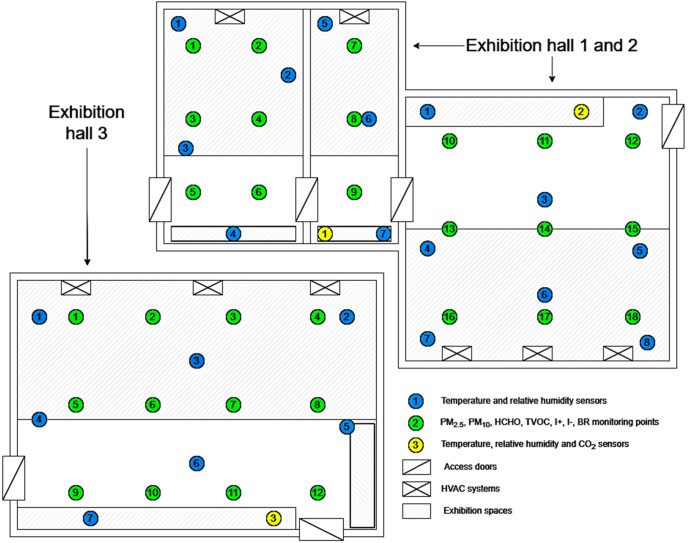
Spatial distribution of datalogger measurement devices and data collection points at the level of the three analyzed rooms inside Țării Crișurilor Museum.

Data collection was done both with datalogger sensors, in order to better capture the variations of the analyzed indicators, and with manual pollutant detectors. As for the datalogger sensors, they were set to automatically take and store information regarding the IAQ indicators every hour, being placed in strategic points in order to have the most accurate measurements and coverage of the rooms. The datalogger systems were used to determine the values of T, RH, CO_2_, PM_2.5_, PM_10_, TVOC and HCHO continuously and simultaneously during the 8 months. In addition to these devices, manual ones were also used, especially due to the fact that for the indicators I^+^, I^-^, AL, PM_2.5_, PM_10_ it was desired to obtain values from several points within the museum, in order to obtain the widest possible coverage and create an adequate database, which would allow analysis from a spatial and comparative perspective. The measurement frequency for these indicators ranged between one and three determinations per day, depending on the availability and accessibility of the monitored spaces. For each determination, the sampling duration was set to one minute per parameter, and the reported value represents the mean obtained over this interval. It should be noted that for these parameters the measurements were not performed simultaneously, but sequentially, depending on the availability of the instruments and operators. The distribution of datalogger device location points and manual value collection points is presented in Fig 3.

For monitoring T and RH, considering that they are very important indicators for the preservation of the exhibits, 22 KlimaloggPro thermohygrometers and HOBO U23 Pro v2 datalogger sensors were used. These were distributed as evenly as possible throughout the rooms to accurately capture any variation in T and RH. CO_2_ was determined at three distinct points, one Extech SD800 datalogger device being placed in each of the three rooms, on the routes frequented by tourists, approximately 1.7 m above the ground, in order to simulate as well as possible the average breathing level of a human. As in the case of CO_2_, for determining PM_2.5_ and PM_10_, three DeltaOHM HD50PM datalogger devices were used, positioned at 1.7 m height in each of the three exhibition halls. HCHO and TVOC were determined using Evikontroll Gas detection and control system devices equipped with specialized sensors for recording the two indicators (Table 1).

**Table 1:** The devices used for data acquisition, along with their technical specifications and usage methodology.

Device model	Producer	Measured pollutants	Accuracy	No. of positions	Datalooger
KlimaloggPro thermohygrometers	TFA, Ottersberg, Germany	T and RH	±1°C, ± 3%	22	✓
HOBO U23 Pro v2	Onset Computer Corporation, Bourne, Massachusetts, USA		±0.2°C, ± 2.5%		✓
DeltaOHM HD50PM	Senseca Italy Srl, Padova, Italy	PM_1_, PM_2.5_, PM_4_, PM_10_	±10 µg/m^3^ (0–100 µg/m^3^) ±10% µg/m^3^ (100–1000 µg/m^3^)	3	✓
Evikontroll Gas detection and control system devices	Evikontroll Gas, Tartu, Estonia	T, RH, CO_2_, HCHO, VOC	±0.5°C (T), ± 5% (RH), ± 50 ppm (CO_2_), ± 0.01 ppm (VOC and HCHO)	3	✓
PCE-PCO 2	PCE Instruments UK, Southampton, United Kingdom	PM0.3, PM0.5, PM1, PM2.5, PM5, PM10	±5%	30	×
Extech SDL400	Extech Instruments, Nashua, United States	AL	±4%		×
NKMH-103	Ion Trading, Tokyo, Japan	I+ and I−	Not exactly defined by the manufacturer		×

Manual measurements were carried out at 18 points in Exhibition Hall 1 & 2 (EH 1–2) and 12 points in Exhibition Hall 3 (EH 3), with the aim of determining PM_2.5_, PM_10_ and AL in order to be able to perform spatial distribution analyses, while for I+ and I- measurements were carried out with manual equipment because at the moment there are no fully automated devices for these indicators. All measurements were taken at a height between 1.5 and 1.7 meters, corresponding to the average height of a person, to allow the analysis of the results in relation to possible negative effects on the health of employees and visitors (Table 1).

Regarding PM, this study focused on PM_2.5_ and PM₁₀, due to their major importance on IAQ and human health. Although the fine fraction PM₁ (particles with an aerodynamic diameter of less than 1 micron) is also relevant, PM_2.5_ and PM₁₀ were prioritized because they are standard indicators for particle pollution and are subject to air quality regulations. In addition, measuring PM_2.5_ and PM₁₀ is more feasible in most indoor environments because instruments for these fractions are accessible and easy to use, allowing building personnel to perform periodic daily or as-needed monitoring.

The data obtained were subjected to rigorous analysis, both in terms of individual values and trends. The statistical analyses were descriptive and comparative, including the determination of mean, minimum and maximum values, standard deviation, dispersion degree and spatial distributions of the monitored indicators. Data processing and visualization were performed using the ArcGIS Pro (spatial analyses and thematic mapping), R 4.3.2 (descriptive statistical analyses and graphical representations) and MATLAB 9.7 (modeling and interpretation of data series).

Permission to conduct this study within the museum was obtained from the institution’s management.

### 3.2. Assessment of heritage microclimatic risk and human microclimatic risk

To identify the risk to which the building and the objects inside are exposed due to the action of the microclimate, the application of the HMR index was chosen, as explained in detail in the works of Bonora et al. [40], Schito et al. [41], and Sharif-Askari and Abu-Hijleh [7]. This is an index that indicates the degree of internal risk, taking into account numerous indicators of the indoor microclimate, as well as numerous international standards in force. Therefore, there are no fixed indicators and standards that must be considered for the achievement of this index, but they are flexible, depending on the nature of the materials inside, the available database and the needs of the project [9]. Consequently, the more environmental indicators are integrated into the calculation of the HMR index, the more comprehensive and reliable the assessment obtained is, providing a closer representation of real microclimatic conditions and their associated risks.

This HMR index can be developed and adapted for a wide range of purposes, depending on the specifics of the environment analyzed. Within the framework of this study, the development of an index dedicated to indoor spaces with exhibits (HMRE), aimed at assessing the suitability of air quality for their conservation, as well as a complementary index (HMRH) intended to determine the impact on the health of visitors and employees, was pursued. First, the implementation of HMRE was considered, taking into account the two components that define it, HMR_env_ (HMR – average) and HMR_osc_ (HMR – oscillation).

*HMRenv* indicates the average level of microclimatic risk, determined based on the average values of the monitored environmental parameters, compared to the limits established by the international standards in force.

*HMRosc* measures the variations (oscillations) of microclimatic parameters over time, which can generate physico-chemical stress on the materials. Thus, even if the average (HMRenv) falls within the standards, too large variations can cause degradation for the exhibits.

Therefore, HMR_E_ can be expressed mathematically as follows (Fig 2):


$${HMR}_{E}=\frac{\left({HMR}_{env}+{HMR}_{osc}\right)}{\text{2}}$$ (1)

where HMR_env_ is calculated according to Formula (2):


$${HMR}_{env}=\text{1}-\left[\left(\frac{{HMR}_{e.high}-{HMR}_{env.data}}{{HMR}_{e.high}-{HMR}_{e.low}}\right)\times \text{2}\right]$$ (2)

where *HMR*_*e.high*_ is the maximum value allowed by the international standards in force taken into account, multiplied by (1 + the oscillations considered allowed by the standard, expressed in percentages), *HMR*_*e.low*_ is the minimum value allowed by the international standards in force taken into account, multiplied by (1 − the oscillations considered allowed by the standard, expressed in percentages), and *HMR*_*env,data*_ is given by the relationship (*M*_*env,data*_*/N*), where *M*_*env,data*_ is the sum of the indicator values calculated for the entire monitoring period for each variable and *N* represents the number of values recorded for the entire monitoring period for each variable [9,40,41].

HMR_osc_ is calculated according to Formula (3):


$${HMR}_{osc}=\text{1}-\left[\left(\frac{{\Delta osc}_{high}-{HMR}_{osc,data}}{{\Delta osc}_{high}-{\Delta osc}_{low}}\right)\times \text{2}\right]$$ (3)

where *Δosc_high_* represents the upper limit of the daily oscillations allowed, according to the applicable international standards, adjusted by multiplying by (1 + the percentage of variation allowed by the standard). Similarly, *Δosc_low_* corresponds to the minimum value allowed for daily oscillations, multiplied by (1 − the percentage of variation allowed). The value of *HMR*_*osc,data*_ is determined by the ratio *ΔM*_*osc,data*_*/N*, where *Δ*_*Mosc,data*_ represents the sum of the oscillations recorded over the entire monitoring period for each parameter analyzed (according to formula 4), and *N* is the total number of values recorded in the same period for the respective variable.


$$\begin{array}{c} {\Delta M}_{osc,data}=\sum {\mathrm{\backslash nolimits}}_{k=\text{1}}^{n}\sum {\mathrm{\backslash nolimits}}_{j=i}^{\text{2}\text{4}}\left(X_{day,k,hourj}-X_{day(k+\text{1}),hourj}\right) \end{array}$$ (4)

where *X* is the variable considered, *k* is the number of days in the monitoring period, and *j* stands for the daily hours (from 00:00 to 24:00) [42].

**Fig 4:**
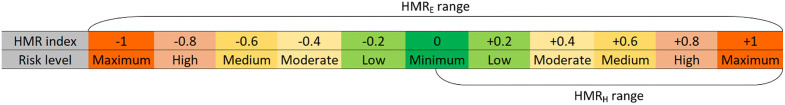
Risk levels associated with the internal microclimate based on the HMR_E_ and HMR_H_ indices, according to the model proposed by Fabbri and Bonora [42].

In this study, the values of T, RH and AL were used to calculate the HMR_E_ index, these three parameters being considered the most influential on the stability and conservation of sensitive materials, such as those made of natural fibers and wood. The evaluation was carried out in relation to the international conservation standards applicable to each parameter. The values obtained were interpreted according to the specific HMR_E_ risk scale, where a score of 0 reflects a minimal risk, indicating optimal conditions, a score close to +1 signals an increased risk generated by exceeding the maximum recommended values, and a score close to −1 signals a risk determined by values below the minimum thresholds allowed for effective conservation (Fig 4) [42]. Considering that for all three indicators, international standards indicate a well-established range of acceptability, the applied methodology considers assigning the indicator 0 to any value that is within the indicated range, and if the values exceed the upper or lower limit, the HMR_H_ increases directly proportionally to the value, number and frequency of exceedances.

The specific HMR formula has been adapted and extended to take into account other parameters of the indoor microclimate, important for ensuring the health of visitors and employees (HMR_H_) (Fig 2). HMRH it is an original extension of the HMR framework, as previously designed by Bonora et al. [40], Schito et al. [41] and Sharif-Askari and Abu-Hijleh [7], and included for the first time in Caciora et al. [9]. Even though the values indicated by the international standards in force for these parameters are not represented by a range, but most of the time by a standard value, a threshold, compared to which the values of the indicators must be lower. In such cases, the risk assessment is carried out depending on the exceeding or non-observance of that limit threshold. Thus, all values that are below the threshold are given the indicator 0 (minimum influence on human health), and those that exceed that limit will be given an indicator closer to 1 depending on the nature of the exceedances, their degree, frequency and number.

In determining the HMR_H_, the HMR_env_ component reflects the degree of proximity or distance of the average value of a parameter from the established threshold, while the HMR_osc_ component evaluates its variations or oscillations in relation to that threshold, including the frequency and magnitude of recorded exceedances. The HMR formula has been adapted to capture both the proportion of time in which the threshold is exceeded and the severity of these exceedances.

In this case, HMR_env_ measures the average risk based on the comparison between the average of the observations and the defined threshold by:


$${HMR}_{env}=\text{1}-\left(\frac{P_{mean}-P_{opt}}{P_{max}-P_{opt}}\right)\times \text{2}$$ (5)

where *P*_*mean*_ is the mean of the pollutant observed during the monitoring period, *Po*_*pt*_ is the optimal value or the minimum threshold below which the values are considered safe, and *P*_*max*_ is the maximum threshold that must not be exceeded.

Regarding HMR_osc_, it can be expressed as:


$${HMR}_{osc}=\text{1}-\left(\frac{{\Delta P}_{max}-{\Delta P}_{mean}}{{\Delta P}_{max}-{\Delta P}_{opt}}\right)\times \text{2}$$ (6)

where *ΔP*_*max*_ is the maximum variation observed within the pollutant, *ΔP*_*mean*_ is the average of the variations for the pollutant, and *ΔP*_*opt*_ is the optimal variation, that is a minimum variation considered safe. In this context, *P*_*opt*_ and *ΔP*_*opt*_ could be considered as minimum or base values from which deviations are measured and *ΔP*_*max*_ represent the upper limits of acceptability [42].

The final HMR_H_ score is expressed as the arithmetic mean of HMR_env_ and HMR_osc_:


$${HMR}_{H}=\frac{{HMR}_{env}+{HMR}_{osc}}{\text{2}}$$ (7)

where


$${HMR}_{H}=\left[\left(\text{1}-\frac{P_{mean}-P_{opt}}{P_{max}-P_{opt}}\times \text{2}\right)+\left(\text{1}-\frac{{\Delta P}_{max}-{\Delta P}_{mean}}{{\Delta P}_{max}-{\Delta P}_{opt}}\times \text{2}\right)\right]$$ (8)

To carry out these assessments, the reference thresholds established by the international standards in force, corresponding to each indicator analyzed, were used. As for pollutants, the analysis focused on the values obtained for HCHO, TVOC, CO₂, I⁺ and I ⁻ , as well as for PM_2.5_ and PM₁₀. According to the method applied, an HMR_H_ index close to zero indicates a reduced risk to the health of visitors and staff, while values closer to 1 suggest an increased level of exposure and, implicitly, a greater risk to human health. In the case of HMR_H_, unlike HMR_E_, the reporting scale does not include negative values (from 0 to −1), due to the fact that the parameters taken into account do not induce stress due to low values, but due to exceeding a certain threshold indicated by international standards; therefore, the exceedings can only be positive, and the index only between 0 and +1 (Fig 4).

For both indices, HMR_E_ and HMR_H_, the same monitoring database was used, thus ensuring the coherence and comparability of the results. The HMR_env_ component was calculated based on all hourly measurements, taking into account the specific indicators of each index (T, RH and AL for HMR_E_, respectively T, RH, CO₂, PM₂.₅, PM₁₀, HCHO, TVOC, I^+^ and I^+^ for HMR_H_). The HMR_osc_ component was determined from the same data, but aggregated over 24-hour intervals, to assess the amplitude of microclimatic fluctuations associated with these parameters.

### 3.3. Antibacterial treatment of the yarns and nanoparticles application

To perform microbiological tests and evaluate the applicability of the AgNPs treatment, six material samples were collected from the exhibits inside the museum (Fig 2).

The fabrics proposed for analysis are part of the textile collection of the ethnography section, having the quality of ethnographic museum objects and subject to the norms of preservation, conservation and valorisation according to the legal provisions in force. They are textiles specific to the Crișul Alb River basin villages and made between the end of the 19^th^ century and the first half of the 20^th^ century. They are made of threads of vegetable and animal origin, namely hemp, cotton and wool (among the most sensitive to biological contamination and microclimatic degradation), processed in the peasant household with traditional means. They are part of the category of blankets and bed covers. The six microbiological samples were selected as reference materials for the ethnographic collection, as they reflect the typological characteristics and vulnerabilities of the traditional textiles exhibited in the collection, representative for assessing the risk of biological contamination and the efficiency of the treatments applied.

To evaluate the effectiveness of the antibacterial treatment applied to textile fibers, an experimental protocol was implemented that included the prelevation of 6 samples from the natural fibre exhibits, the treatment of samples with AgNPs and microbiological analysis using standardized methods. The methodological steps aimed at both the application of the treatment and the monitoring of antimicrobial activity over time (Fig 2).

For the antibacterial treatment, Rucco-Bac AgNPs supplied by Francotex (Cocentaina, Spain) were used. In addition, for pH adjustment, it was necessary to use Citric Acid supplied by Sigma-Aldrich (Barcelona, Spain). For microbiological analysis, Petrifilm™ plates were used for counting aerobic bacteria (Aerobic Count, AC). The plates used in this study are ready-to-use culture media, composed of nutrients from Standard Methods Agar, a cold-water-soluble gelling agent, and a red indicator dye that facilitates the visualisation and counting of colonies. Petrifilm™ AC plates were obtained from the company 3M™. For the dilution medium used in the microbiological assay, bacteriological peptone supplied by Cultimed was used.

The antibacterial treatment was carried out by dispersing the product (30 g/L) in distilled water. The pH was adjusted to 4.5–5 by the addition of citric acid. The yarn samples were immersed in the dispersion for 5 minutes and then passed through a filter, ensuring a pick-up between 70–80%. In order to carry out inoculation on the Petrifilm plates, a diluent and suspension medium is required for the samples, such as peptone water. Peptone water provides a suitable environment for the recovery and maintenance of bacterial viability prior to plating and also serves as a medium to dilute the sample to obtain an appropriate concentration of microorganisms for counting. A concentration of 10 g/L of bacteriological peptone in distilled water was used for its preparation. The solution was brought to the boil and maintained for several minutes to achieve sterilisation.

The dilution used was 1:50, calculated based on the sample’s weight (fibre weight). Subsequently, 1 mL of each dilution was inoculated onto Petrifilm™ AC plates, which were incubated in an oven at 35°C for 72 hours. After the incubation period, plate counting and analysis were carried out, considering the dilution used (1:50).

To calculate the Colony Forming Units (CFU) per gram (g) of the original sample, the following formula was used:


$$\text{CFU}/\text{g}=\frac{NCC\times DF}{VP}$$ (9)

where *NCC* stands for the number of colonies counter, *DF* is the dilution factor and *VP* is the volume plated in mL (1 mL).

To assess the persistence of the antibacterial effect, all six analyzed samples were subjected to two successive microbiological tests, performed at an interval of 30 days. It should be noted that, after the first analysis, the samples were not dried, but were kept with the peptone water residue, in order to favor possible bacterial growth for a correct assessment of the treatment efficiency.

## 4. Results

### 4.1. The determination of the indoor air quality

**Fig 5:**
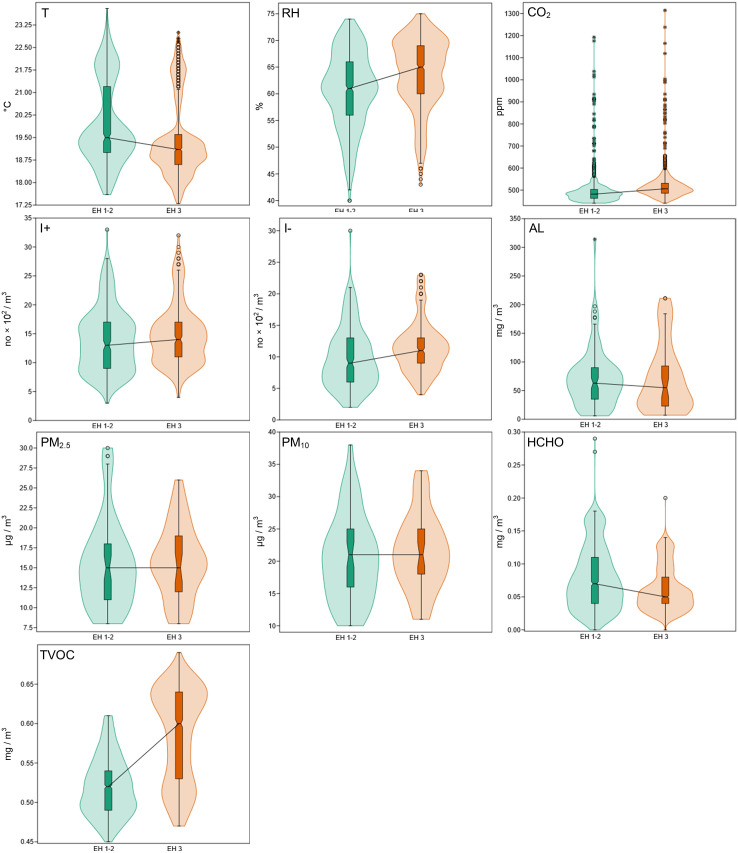
Values obtained for the 10 indicators of the IAQ for the conservation of exhibits and ensuring human health.

The T maintains an average of 19.7°C with minimal room-to-room variations since EH 1–2 averages 19.9°C with 1.38°C std. Dev. and EH 3 averages 19.4°C with 1.28°C std.dev. The T dispersion in both rooms remains moderate with 6.2°C in EH 1–2, between 23.8°C and 17.6°C, and 5.7°C in EH 3 between, 23°C and 17.3°C. The T remains stable throughout all analyzed rooms because there are no significant T fluctuations that could compromise exhibit preservation. A similar situation is recorded in the case of RH, which had an average value of 60.8% and a std. dev. of 7.08% in EH 1–3 and 63.9% with std. dev. 6.56% in EH 3. The dispersion in the case of EH 1–2 is 31%, with maximum values of 71% and minimum values of 40%, while in EH 3 the dispersion indicates the value of 32%, with maximum values of 75% and minimum values of 43% (Fig 5). For the two indicators, ASHRAE Standards [43] indicate that an average value of 20°C (±1°C–2°C) in the case of T and 50% (±3%) for RH must be maintained; but these can also evolve temporarily within the limits of 16°C–24°C in the case of T and 45–60% in terms of RH. These values present a range in which the respective indicators can evolve without representing a risk to the integrity of the exhibits. The average values of T are maintained within the set multi-annual standard, without presenting a potential risk to the exhibits. The average values of RH are 10–13% above the limit represented by the multi-annual value admitted by the standard, respectively at the limit of acceptability in the case of the wider spectrum of the time-weighted average concentration (up to 60%).

The CO_2_ values had an average of 506 ppm, a value that falls within the international standards in force for ensuring human health, which indicate the value of 1000 ppm as a safety threshold [44]. The average value in the case of EH 1–2 was 493 ppm, std.dev. 67 ppm, with maximum values of 1192 ppm and minimum values of 441 ppm. In EH 3, the values are higher, with an average of 519 ppm, std.dev. of 71 ppm, maximum values of 1314 ppm and minimum values of 440 ppm (Fig 5). Values above 1000 pm were recorded very rarely in the IAQ monitoring campaign, being reported in only 0.4% of cases. This indicates that high levels of CO_2_ are very rare in these rooms, without affecting the health of visitors and staff.

Although there are no international standards for I^+^ and I^-^ concentrations, some relevant studies in the field [45,46] indicate that for human health and for the best possible IAQ, I^+^ should be kept below 1000 ions/cm^3^, while I^-^ should be as high as possible (but above 1000 ions/cm^3^). The recorded values indicate a mean I^-^ value of 1057 ions/cm^3^. In the case of EH 1–2, the mean was 960 ions/cm^3^ (std. dev. of 477 ions/cm^3^), with maximum values of 3000 ions/cm^3^ and minimum values of 200 ions/cm^3^. In EH 3, I^-^ had mean values of 1154 ions/cm^3^ (std. dev. of 545 ions/cm^3^), maximum values of 2300 ions/cm^3^ and minimum values of 400 ions/cm^3^. Regarding I^+^, the average value was 1431 ions/cm^3^, EH 1–2 having an average of 1380 ions/cm^3^, while EH 3 prevailed with a higher average of 1482 ions/cm^3^. The maximum values were 3300 ions/cm^3^ in the case of EH 1–2 and 3200 ions/cm^3^ in the case of EH 3, the minimum values being 300 ions/cm^3^, respectively 400 ions/cm^3^ (Fig 5).

I^+^ exceeded 1000 ions/cm^3^ in 80% of the time, which indicates their supersaturation in the air. A high level of I^+^ can contribute to fatigue, stress, decreased concentration and a more electrostatically charged air, which is not beneficial for the quality of the indoor environment. I^-^ values were above 1000 ions/cm^3^ 55.8% of the time, which shows that they are present in a more balanced, but not consistently optimal proportion.

The HCHO concentration had an overall average of 0.07 mg/m^3^ in the museum, EH 1–2 had an average of 0.078 mg/m^3^ (std. dev. 0.047 mg/m^3^), and EH 3 stood out with an average of 0.062 mg/m^3^ (std. dev. 0.033 mg/m^3^). In EH 1–3 the values amounted to a maximum of 0.29 mg/m^3^ and a minimum of 0.004 mg/m^3^, while in EH 3 they were 0.2 mg/m^3^ for the maximum and 0.00 mg/m^3^ for the minimum. According to international standards [47], multiannual concentrations of HCHO in indoor air should not exceed 0.04 mg/m³, in order to avoid any adverse effects on human health. The average of this indicator is above the permitted limit, and 71.7% of the values related to EH 1–2 and 62.8% of those recorded in EH 3 exceed the limit allowed by the international standards in force, which may indicate long-term exposure above the optimal level, which can affect human health.

The World Health Organization [48] indicates for TVOC a multiannual permissible concentration that should not exceed 1 mg/m^3^. Prolonged exposure to concentrations higher than 1 mg/m^3^ of TVOC can lead to respiratory and eye irritation, headaches and decreased concentration capacity, thus negatively affecting human health [49]. The recorded values show an overall average of 0.55 mg/m^3^, with a value of 0.52 mg/m^3^ (std.dev 0.037 mg/m^3^) in the case of EH 1–2, respectively 0.59 mg/m^3^ (std. dev. 0.059 mg/m^3^) in EH 3. The maximum values were 0.61 mg/m^3^ (EH 1–2) and 0.69 mg/m^3^ (EH 3), and the minimum values indicated 0.45 mg/m^3^ and 0.47 mg/m^3^. These concentrations are comfortably below the recommended threshold of 1 mg/m³, indicating good IAQ from a TVOC perspective; no value recorded exceeded the international standard.

**Fig 6:**
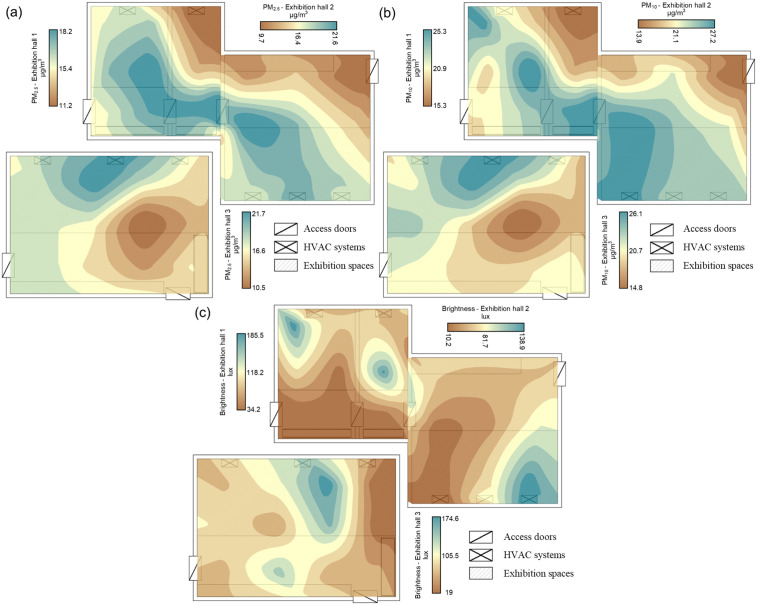
Spatial distribution of the average values of the indicators measured at the level of the three rooms in the study. (a – spatial distribution of PM2.5; b – spatial distribution of PM10; c – spatial distribution of luminosity).

The evaluation of PM_10_ and PM_2.5_ values between the rooms reveals substantial distinctions. The recorded PM_10_ values in EH 1–2 show moderate variability, with an average of 20.4 µg/m³ while std. dev. reaches 6.57 µg/m³. The lowest measurement reached 10 µg/m³, but the highest measurement exceeded 38 µg/m³, which indicates brief periods of high particle concentration. The PM_2.5_ measurements in this room average 15.5 µg/m³ with a std. dev. of 5.52 µg/m³. The recorded values span from 8 µg/m³ to 30 µg/m³. The PM_10_ concentration in EH 3 reaches 21.3 µg/m³, which is higher than EH 1–2 but has a lower std. dev. of 5.82 µg/m³. The recorded values in this room ranged from 11 µg/m³ to 34 µg/m³, indicating a variation interval of 23 µg/m³, which reflects a more uniform distribution than in the other room. The PM_2.5_ measurements in EH 3 show a mean of 15.67 µg/m³ with a standard deviation of 4.52. The PM_2.5_ concentrations in EH 1–2 ranged between 8 µg/m³ and 26 µg/m³, with a variation interval of 18 µg/m³, suggesting smaller fluctuations compared to EH 1–2 (Fig 6).

The PM_2.5_ indicator showed its highest values in the central part of EH 1–2, with measurements between 18.2 µg/m³ and 21.6 µg/m³, while EH 3 recorded the highest PM concentrations in its exhibition area, with a maximum average of 21.7 µg/m³ (Fig 6a). The PM_10_ distribution across the two rooms showed similar patterns, but EH 1–2 reached its highest average at 25.3 µg/m³ and 27.2 µg/m³ while EH 3 reached its highest at 26.1 µg/m³; the lowest values remained at 13.9 µg/m³ (EH 1–2) and 10.2 µg/m³ (EH 3) (Fig 6b). As a general rule regarding the distribution of PM_2.5_ and PM_10_, their values are higher near HVAC systems. This may be due to particle recirculation, particle input from outside, mechanical wear of HVAC components (especially filters) and lack of proper maintenance.

All averages recorded over the entire monitoring period for PM_10_ and PM_2.5_ exceed the multi-annual value of 12 µg/m³ set by the international standards in force [50]. On the other hand, no monitoring days were recorded averages that exceeded the daily permissible threshold of 35 µg/m³.

Maintaining an optimal brightness value is essential for the good conservation of exhibits, especially those prone to fine deterioration (paper, fibres, etc.). Within the two exhibition halls, the average AL was 71.8 lux, with values of 70.5 lux in the case of EH 1–2 and 73 lux in EH 3. The EH 1–2 area reached its highest illumination at 314 lux while its lowest point reached 6 lux, and EH 3 experienced its peak at 211 lux and its minimum at 7 lux (Fig 6). The British Standards Institution [51] specifies that rooms containing valuable objects should maintain illumination levels between 50 and 200 lux for optimal conservation. The degradation process of materials and exhibits in heritage buildings accelerates when illumination exceeds these established values. In this case, the averages of the two rooms fall within this standard; the individual situations in which the amount of AL exceeds the upper limit of the standard were 9.6% in the case of EH 1–2 and 6.7% in the case of EH 3. Regarding the spatial distribution of AL in the three analysed rooms, it is observed that the distribution is uneven, being higher in the exhibition spaces than in the transit ones. Thus, the maximum average value amounts to 185.5 lux and 138.9 lux in the case of EH 1–2 and to 174.6 lux in the case of EH-3 (Fig 6c).

### 4.2. Assessment of heritage microclimatic risk and human microclimatic risk

The results obtained for the HMR_E_ and HMR_H_ indexes are based on the working methodology presented previously and on the values indicated in the international standards in force for each parameter, presented in detail in subchapter 4.1. Determination of indoor air quality and in Table 2.

**Table 2:** Thresholds and ranges recommended by the international standards in force used for calculating HMR_E_ and HMR_H_ values.

Indicator	Threshold/Ranges		International standards
	HMR_E_	HMR_H_	
T	16-24°C	×	ASHRAE Standard [43]
RH	45-60%	×	
AL	50-200 lux	×	British Standards Institution [51]
CO_2_	×	≤ 1000 ppm	ANSI/ASHRAE Standard [44]
HCHO	×	≤ 0.04 mg/m³	EPA Standard [47]
TVOC	×	≤ 1 mg/m^3^	World Health Organization [48]
PM_2.5_	×	≤ 35 µg/m³	EPA Standard [50]
PM_10_	×		
I^+^	×	≤ 1000 ions/cm^3^	Jayaratne et al. [45], Renye et al. [46]
I^-^	×	≥ 1000 ions/cm^3^	

For HMR_E_, the values obtained were 0.39 for EH 1–2 and 0.41 for EH 3, with an overall average value of 0.4 (Table 3). These results correspond to a moderate degree regarding the risk of degradation that indoor air poses to museum exhibits. This moderate risk stems mainly from the fact that RH values are above the limit allowed by ASHRAE Standards [43], indicated by HMR_env_ with a value of 0.56 in the case of EH 1–2 and 0.73 in EH 3. At the same time, RH values fluctuate within a fairly extensive range, above the upper limit of 60%, so that HMR_osc_ values are recorded as 0.9 on average.

**Table 3:** The results obtained from the application of the HMR_E_ indicator within the three analysed rooms of the Țării Crișurilor Museum.

	Exhibition hall 1 & 2				Exhibition hall 3				Overall average
Inidcators	T	RH	AL	Room average	T	RH	AL	Room average	
HMR_env_	0.14	0.56	−0.35	0.12	0.1	0.73	−0.38	0.15	0.14
HMR_osc_	0.47	0.88	0.66	0.67	0.37	0.92	0.72	0.67	0.67
HMR_E_	0.3	0.72	0.15	0.39	0.24	0.82	0.16	0.41	0.4

Added risk is also determined by the T indicator, even if its values remain within acceptable limits, HMR_env_ obtaining only 0.14 in the case of EH 1−2 and 0.1 in the case of EH3. The oscillations of this indicator above the upper limits determined by the standards in force are non-compliant, although these are not very frequent or large; HMR_osc_, in the case of T, obtains only an average value of 0.4. In contrast, lower values were recorded for AL, with only 0.15 in EH 1−2 and 0.16 in EH 3 regarding the HMR_E_ indicator. The exceedances of this indicator exceeded the lower limit of acceptability, according to the British Standards Institution [43], which is why HMR_env_ recorded negative values in all rooms, with an average of −0.37 (Table 3).

However, these determinations only provide a theoretical estimate of the potential for degradation that the analysed indicators may have on the monument and the exhibits. They do not reflect the entire spectrum of real risks, since the degree of deterioration is also influenced by the combined action of multiple factors, which may interact in a complex manner. Among these are phenomena that are difficult to quantify, such as the presence of biological agents, the composition of the exposed materials, or the interaction of pollutants with sensitive surfaces.

The overall HMR_H_ risk value, calculated as an average for both rooms, was 0.128, with values of 0.14 within EH 1–2 and 0.11 within EH 3. The HMR_env_ indicator had overall average values of 0.171 and HMR_osc_ of only 0.084 (Table 4). These results indicate that the air inside the analysed exhibition spaces is favourable for human activity and presents a low risk.

**Table 4:** The results obtained following the application of the HMR_H_ indicator within the three analysed rooms of the Țării Crișurilor Museum.

	Exhibition hall 1 & 2								Exhibition hall 3								OA
Inidcators	CO_2_	HCHO	TVOC	PM_2.5_	PM_10_	I^+^	I^-^	RA	CO_2_	HCHO	TVOC	PM_2.5_	PM_10_	I^+^	I^-^	RA	
HMR_env_	0	0.951	0	0	0	0.38	0.04	0.195	0	0.551	0	0	0	0.48	0	0.147	0.171
HMR_osc_	0.0018	0.167	0	0	0.0185	0.198	0.259	0.092	0.0021	0.158	0	0	0	0.229	0.142	0.075	0.084
HMR_H_	0.0009	0.56	0	0	0.0093	0.29	0.15	0.144	0.0011	0.35	0	0	0	0.36	0.07	0.111	0.13

RA – room average; OA – overall average.

**Fig 7:**
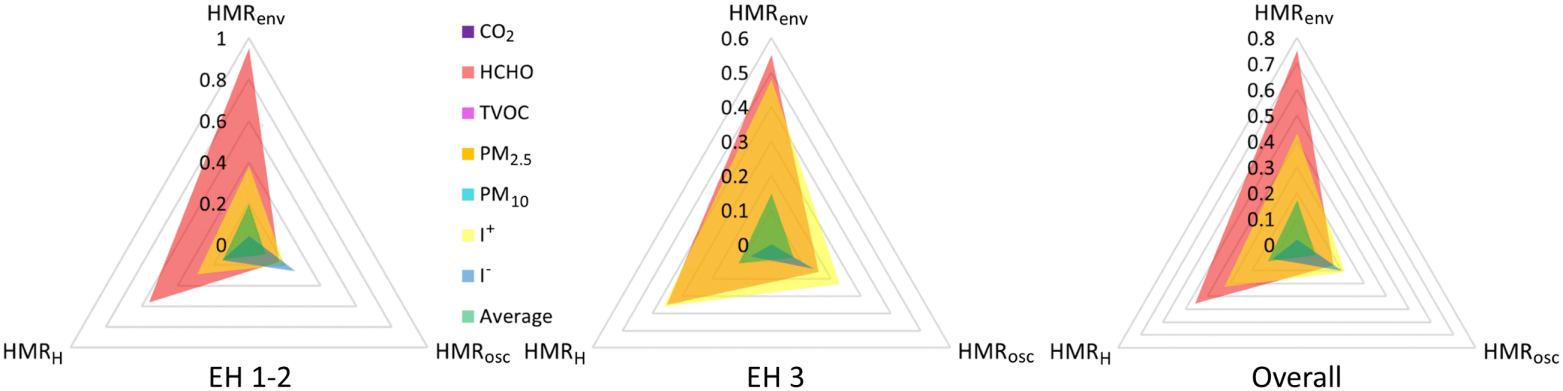
Values obtained for each pollutant considered for the construction of HMR_H_ within EH1-2 and EH 3.

The vast majority of the analysed indicators obtained relatively low values, both for HMR_env_ and HMR_osc_ and finally for the combined HMR_H_ index. However, some indicators with potentially harmful effects to human health were also recorded. Thus, the highest values were obtained by HCHO, which in EH 1–2 recorded a value of 0.951 in the case of HMR_env_, respectively 0.551 in EH 3 and a combined HMR_H_ index of 0.46. This indicates that HCHO frequently and vastly exceeded the international standards in force, although its oscillations are relatively small during the monitoring period (HMR_osc_ of 0.163). Higher values were also recorded by I + , with HMR_H_ of 0.33, HMR_env_ of 0.43 and HMR_osc_ of 0.21. At the same time, I had values below the standard values of 1000 in some situations, obtaining values of 0.11 of the HMR_H_ indicator, 0.02 in the case of HMR_env_ and 0.20 in the case of HMR_osc_ (Fig 7 and Table 4).

The rest of the analysed indicators (CO_₂_, TVOC, PM_2.5_ and PM₁₀) fall within the limits established by current international standards, recording HMR_H_ index values below 0.1. Remarkably, for TVOC and PM_2.5_, the HMR_H_ value was 0, indicating the total absence of risk to human health (Fig 7 and Table 4). These results highlight the existence of a stable and safe indoor microclimate from a sanitary point of view.

### 4.3. Antibacterial treatment of the yarns and nanoparticles application

Antibacterial tests were carried out on the original samples and those treated with AgNPs, a well-known antimicrobial agent. This analysis aims to identify whether the yarns exhibit natural antibacterial activity or if additional treatment is necessary to enhance this property.

Table 5 shows the colony forming units (CFU/g) for each sample at two different times: Test 1 (immediately after nanoparticle treatment) and Test 2 (after 30 days from Test 1). Most untreated samples in Test 1 showed significant antimicrobial activity with low CFU values or no detectable colonies (samples 2, 3 and 6). It is suggested that antibacterial compounds may be derived from natural dyes in the textile yarns.

**Table 5:** Aerobic bacterial count. Colony-forming units (CFU) per gram of sample.

	Test 1		Test 2 (30 days after Test 1)	
Sample	No Antibacterial Treatment CFU/g	With Antibacterial Treatment CFU/g	No Antibacterial Treatment CFU/g	With Antibacterial Treatment CFU/g
1	600	150	Uncountable	< 10
2	< 10	100	450	150
3	< 10	100	1350	100
4	200	< 10	Uncountable	< 10
5	250	150	5350	100
6	< 10	200	< 10	100

The results from AgNPs-treated samples also showed low CFU values, which confirms their documented ability to inhibit bacterial growth. In some cases, the differences between untreated and treated samples in Test 1 are minor (e.g., sample 6), which could indicate a native antibacterial activity close to that induced by treatment.

Test 2, performed after 30 days of exposure at room temperature, shows significant differences between untreated and treated samples. The untreated samples, especially 4 and 5, showed a marked proliferation of bacterial colonies (up to 5350 CFU/g), indicating a loss of initial efficiency or favoring microbial development in humid conditions. The treated samples maintained a very low bacterial load, indicating the persistence of AgNPs antibacterial effect over time.

**Fig 8:**
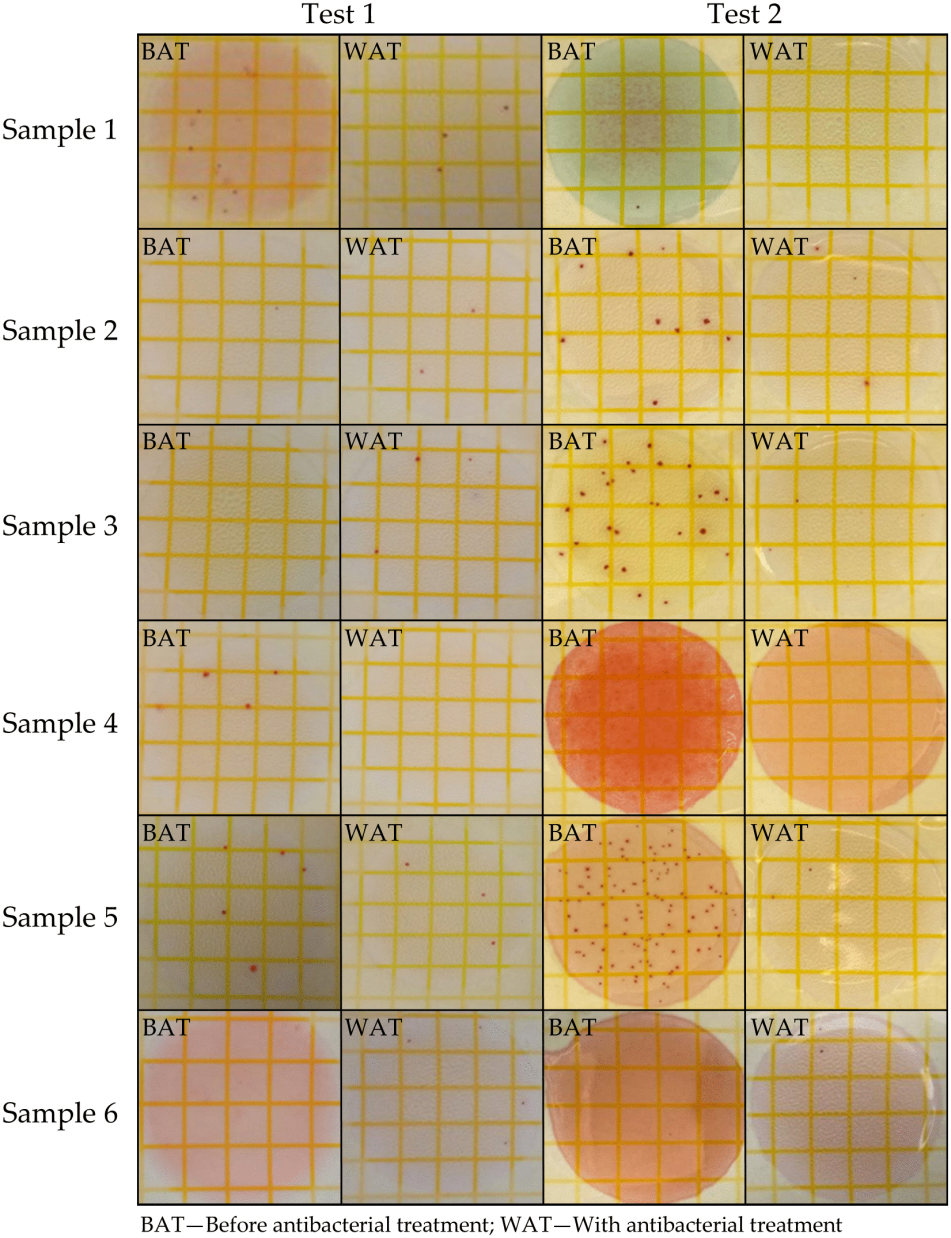
Visual microbiological analysis of the six samples, before treatment, immediately after application of AgNPs and 30 days after treatment.

The bacterial contamination levels in AgNPs-treated samples remained at the same low levels as the initial treatment results. These samples’ lack of bacterial colonies demonstrates that the antimicrobial treatment remained effective throughout the evaluation period. The microbial load differences between treated and untreated samples become evident through the visual representation shown in Fig 8.

**Fig 9:**
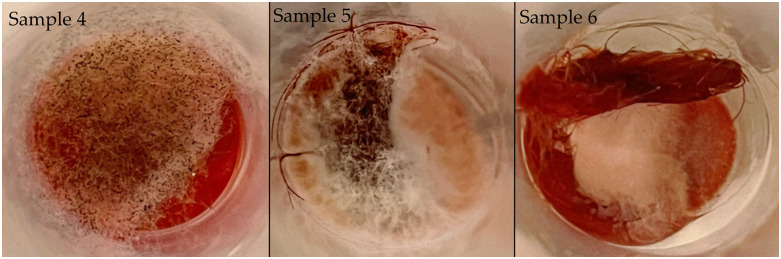
Fungal growth on samples 4, 5 and 6.

The samples were stored at room temperature for thirty days before undergoing their second microbiological assessment. The untreated yarns of samples 4, 5, and 6 displayed fungal growth during visual examination, which indicated their susceptibility to biological contamination when exposed to humid conditions. The second microbiological test revealed elevated bacterial colony numbers compared to the initial analysis, with samples 4 and 5 showing the highest CFU values. The increased microbial growth seems to result from the yarns being exposed to a moist environment, which supports microbial development (Fig 9).

According to this study, the analysed yarns demonstrate substantial antibacterial properties because of the substances used during dyeing. The experimental findings and specialised literature support the conclusion that these yarns received natural dye treatments, which are recognised for their antimicrobial properties. The untreated samples displayed antibacterial properties, which reached levels equivalent to those achieved through AgNPs treatment in certain instances. Natural dyes show promise as both colorants and protective agents for developing advanced biological protection textiles.

The results obtained from the analysis of the antibacterial effect indicate that the investigated textile yarns could be dyed with dyes of natural origin, given the antimicrobial activity observed in the untreated samples.

## 5. Discussion

The results obtained for T and RH values in the three Exhibition Halls analysed indicate that these parameters frequently fluctuated above the thresholds established by the standards in force [43]. Such fluctuations create unstable microclimatic conditions that promote condensation phenomena and increased surface moisture. Under these conditions, significant adverse effects can occur on the conservation of exhibits. High values, associated with frequent fluctuations, favour the development of mould and bacterial colonies, especially on organic materials, such as textiles, wood or paper [52]. This process accelerates both biological degradation (through enzymatic and microbial activity) and chemical deterioration (hydrolysis and oxidation), ultimately leading to fibre weakening, loss of elasticity, and alteration of the original colour or structural integrity of the objects [53,54]. As Ahmed et al. [55] indicated, this phenomenon becomes even more dangerous in the case of naturally dyed textile objects, since plant compounds can react with excess RH and variable T, accelerating the deterioration processes. In this context, applying antibacterial treatments based on AgNPs has proven to be an effective solution for the long-term protection of textile materials exposed to unstable microclimatic conditions. Ilies et al. [29] demonstrated the efficiency of impregnating traditional textiles with AgNPs suspensions, achieving a reduction of over 95% of bacterial colonies, without affecting the structure of the treated material. Also, Lite et al. [56] highlighted that ecologically synthesised AgNPs can be successfully applied to cotton and wool, providing antimicrobial protection of over 99% against pathogens specific to the museum environment. The effectiveness of AgNPs is explained by their ability to disrupt microbial cell membranes, generate reactive oxygen species, and interact with essential biomolecules, leading to the inhibition of microbial growth and colony formation. These mechanisms make AgNPs particularly suitable for the conservation of organic heritage materials, where conventional chemical biocides may be less sustainable or more damaging to the fibres [56].

Regarding protecting human health in exhibition spaces, most monitored parameters do not exceed the safe thresholds. Still, specific indicators have remained at high values. These can affect human health even when all other parameters are within normal limits [10,57].

HCHO recorded very high values, with an average of 0.07 mg/m^3^, while the EPA Standard [47] indicates an acceptability threshold of 0.04 mg/m^3^. Exposure to HCHO, even at low levels, has been associated with irritation of the mucous membranes (eyes, nose, throat), headaches and neuropsychiatric symptoms, such as decreased motor activity, fatigue, general malaise and cognitive impairment in poorly ventilated indoor spaces [58,59]. The frequent exceedances can be attributed to the fact that the building has been recently renovated. It is established that modern building materials, especially those used for insulation, such as varnishes, paints, adhesives and furniture, can be essential sources of HCHO. This is especially true in spaces where T and RH are high, as was found in the museums analysed [60,61]. This may also originate in the cleaning products used [62].

I- values were sometimes below international standards, and I+ frequently exceeded these thresholds during the monitoring period. An imbalance in the concentration of air ions alters the natural ion ratio, which affects the regulation of physiological processes and the perception of indoor air quality. Such situations can cause, among others, thermal discomfort and physiological stress, by disturbing the respiratory function and mucosal balance, as well as respiratory problems and irritations, and may also contribute to decreased cognitive performance through reduced oxygen uptake efficiency and increased fatigue [63]. This instability is caused by the absence of natural ventilation, given that the space has no openings to the outside, and ventilation depends exclusively on the HVAC systems. Studies show that the lack of natural ventilation favours the accumulation of pollutants and microclimatic variability [64], and HVAC systems not optimised for conservation can amplify these fluctuations, negatively affecting the exposed heritage [65].

Considering the values of the pollutants analysed and the time spent indoors (between 1 and 3 hours), visitors’ health is not endangered during their stay. Abelsohn and Stieb [66] argue that, in the short term, such an indoor environment can only accentuate pre-existing conditions, without causing the appearance of new ones. As for employees, even if they spend a long time indoors, and some values of the indoor parameters are not compliant, taking into account that the HMR_H_ indicates very low values (only 0.13 on average), this type of indoor microclimate can cause, in the worst case, a slight discomfort, which usually disappears shortly after leaving the building [67]; especially only in sensitive people and those with pre-existing conditions. However, as also emphasized by Pironti et al. [68], constant monitoring of pollutant levels is recommended, and if the situation requires it, air purification devices can be installed.

## 6. Conclusions

IAQ can significantly influence the conservation of museum exhibits, but it can also represent a risk to the health of visitors, employees, and restorers. The results obtained in the present study indicate a moderate risk generated by IAQ on the exhibits in the Țării Crișurilor Museum. Thus, the HMR_E_ index had an overall value of 0.4, calculated based on the three analysed parameters. The detailed analysis shows that the most significant contribution to this risk is made by RH, which recorded an HMR_env_ score of 0.65 and an HMR_osc_ of 0.9. This high RH can be problematic for conservators, as it can accelerate the development of mould and the degradation of organic materials. The HMR_H_ index indicated a safe environment for human activity, obtaining an overall score of only 0.15, which is attributed to a low-risk environment. However, better management of HCHO, the leading indicator with potential harm to human health, should be considered. At the same time, I^-^ registered relatively low values, and I^+^ high, indicating improperly ventilated and electrostatically charged indoor air, leading to increased fatigue, concentration problems and decreased cognitive capacity.

Applying antibacterial treatment with AgNPs demonstrated significant efficiency in reducing the microbiological load on the organic textile materials analysed. The test results showed that the treated samples recorded low CFU values, both immediately after application and 30 days after treatment, maintaining a constant low level of contamination. In contrast, the untreated samples showed a sharp increase in bacterial and fungal contaminationg. This persistence of the antimicrobial effect confirms the potential of using AgNPs as a sustainable and non-invasive solution for protecting textile heritage, especially in museum contexts where IAQ conditions can vary.

The main achievement of this study is the development of an integrated and comprehensive risk assessment framework, capable of capturing the full spectrum of indoor environmental conditions in museum spaces. Unlike traditional approaches, which focus exclusively on air quality monitoring for the conservation of exhibits, the proposed method simultaneously integrates human health parameters, fungal microflora analysis and the efficiency of nanoparticle-based treatments, providing a holistic perspective on microclimatic stability and safety. The results confirm the initial hypothesis that deviations of key parameters from optimal values can negatively influence both exhibits and the health of visitors and staff, and the proposed HMR_E_ and HMR_H_ indices are able to record them in an integrated and multidimensional way. The study also demonstrates that nanoparticle treatment represents an effective and sustainable solution for reducing microbiological risks, and that optimizing the HVAC system and controlling internal pollution sources are essential for maintaining a balanced, healthy indoor environment conducive to heritage conservation.

## 7. Limitations and future studies

The limitations of this study are that the monitoring period is only 8 months (January-August period), without covering the entire annual cycle. Thus, seasonal influences on IAQ parameters cannot be fully evaluated. At the same time, monitoring a single museum collection was considered, which does not allow the extrapolation of conclusions to the entire museum. The microbiological evaluation and application of AgNPs were done on a limited number of samples, coming only from exhibits made of textile materials. This leaves room for future research to test other materials (wood, paper, leather, etc.). Although theoretical health risks are presented, no clinical or subjective data were collected to confirm direct effects on visitors or staff.

Additionally, it should be noted that the DeltaOHM HD50PM device used for particulate matter monitoring has an accuracy of ±10 µg/m³ for concentrations below 100 µg/m³, according to the manufacturer’s ISO/IEC 17025-certified specifications (ACCREDIA LAT No. 124). Although this introduces a potential absolute uncertainty, the recorded values remain well below international health and safety thresholds, and the instrument’s calibration ensures reliable comparative and risk-based assessments.

Future studies will aim to accurately determine, at the genus and species level, the bacteriological and fungal contamination in the air and interior surfaces (including exhibits), as well as conduct thermographic analyses and analyses of visitors’ perceptions of the IAQ.

## Abbreviations

IAQ: indoor air quality
T: temperature
RH: relative humidity
PM: particulate matter
HCHO: formaldehyde
TVOC: total volatile organic compounds
I+: positive ions
I-: negative ions
AL: artificial light
HMR: Heritage Microclimate Risk
HMR_E_: HMR for the conservation of exhibits
HMR_H_: HMR for human health
EH 1–2: exhibition hall 1 & 2
EH 3: exhibition hall 3
CFU: colony forming units
AgNPs: silver nanoparticles
HVAC: heating, ventilation, and air conditioning
